# Effect of carvacrol on pulmonary function tests, and total and differential white blood cell counts in healthy volunteers: A randomized clinical trial 

**Published:** 2019

**Authors:** Vahideh Ghorani, Marzie Boskabady, Mohammad Hossein Boskabady

**Affiliations:** 1 *Pharmaciutical Research Center, Mashhad University of Medical Sciences, Mashhad* *, * *Iran*; 2 *Department of Physiology, School of Medicine, Mashhad University of Medical Sciences, Mashhad* *, * *Iran*; 3 *Dental Materials Research Center, School of Dentistry, Mashhad University of Medical Sciences, Mashhad, Iran*; 4 *Neurogenic Inflammation Research Center, Mashhad University of Medical Sciences, Mashhad,* *Iran*

**Keywords:** Carvacrol, Pulmonary function tests, White blood cells, Healthy volunteers

## Abstract

**Objective:**

This is the first study to evaluate the effect of carvacrol on pulmonary function tests (PFT), and total and differential white blood cell (WBC) counts in healthy volunteers.

**Materials and Methods:**

Thirty healthy volunteers were recruited based on the inclusion and exclusion criteria. The subjects were treated with two doses of carvacrol (1 and 2 mg/kg/day) for 1 month. Total and differential WBC counts and PFT were examined before and after the treatment period.

**Results:**

There were no statistically significant differences in terms of total and differential WBC counts between pre and post-treatment with the two doses of carvacrol. Also, results of PFT tests indicated that administration of 1 mg/kg/day carvacrol had no effect on PFT parameters when comparing post-treatment values with pre-treatment values. Treatment with 2 mg/kg/day carvacrol for 1 month increased forced expiratory volume in first second (FEV_1_) (p<0.05). However, the changes in total and differential WBC counts as well as PFT values after 1-month treatment were not significantly different between two groups.

**Conclusion:**

The results of this study indicated that treatment of healthy individuals with 1 and 2 mg/kg carvacrol for 1 month has no negative effects on total and differential WBC counts nor PFT values.

## Introduction

Carvacrol (2-methyl-5-(1-methylethyl)-phenol) is a monoterpenic phenol found in numerous aromatic plants, including *Zataria multiflora* (Can Baser, 2008 ▶; Suntres et al., 2015 ▶), *Thymus vulgaris* (Ebrahimzadeh et al., 2003 ▶), *Origanum vulgare* (Burt, 2004 ▶), *Nigella sativa* (Gholamnezhad et al., 2016 ▶).

*In vitro* and *in vivo* studies indicated several biological and pharmacological activities for carvacrol including antimicrobial, antibacterial (Ravishankar et al., 2008 ▶), antifungal (Ahmad et al., 2011 ▶), antioxidant (Jayakumar et al., 2012 ▶), anticancer (Arunasree, 2010 ▶), hepatoprotective (Suntres et al., 2015 ▶), antispasmodic (Boskabady et al., 2011 ▶) and vasorelaxant properties (Busse and Swenson, 1989 ▶). Carvacrol has also shown preventive effects on tracheal responsiveness, inflammatory mediators and lung pathology in animal models of asthma (Fachini-Queiroz et al., 2012 ▶; Farraj et al., 2003 ▶; Landa et al., 2009 ▶; Boskabady and Jalali, 2013 ▶; Boskabady et al., 2014 ▶; Jalali et al., 2013 ▶; Kianmehr et al., 2016 ▶; Boskabady et al., 2016 ▶) and COPD (chronic obstructive pulmonary disease) (Gholami Mahtaj et al., 2015 ▶; Mahtaj et al., 2015 ▶). Relaxant effects of carvacrol on tracheal smooth muscles in sensitized guinea pigs (Boskabady and Jandaghi, 2003 ▶) were possibly mediated through its inhibitory effect on muscarinic (Boskabady et al., 2011 ▶) and histamine (H1) receptors (Boskabady et al., 2012 ▶) as well as its stimulatory effect on β-adrenergic receptors (Boskabady et al., 2010 ▶).

Acute toxicity studies reported carvacrol’s median lethal dose (LD_50_) in various animals. Results of mutagenicity and cytotoxicity studies indicated that long-term genotoxic effects of carvacrol is weak (De Vincenzi et al., 2004 ▶; Stammati et al., 1999 ▶) while cytotoxic effects of carvacrol can make it an effective antimicrobial agent (Özkan and Erdoğan, 2011 ▶). In spite of investigation of carvacrol effects in laboratory tests and animal studies, scientific data on its clinical effects are lacking (De Vincenzi et al., 2004 ▶).

Thus, the aim of the present study was to evaluate the effect of 1-month administration of carvacrol on pulmonary function tests (PFT), and total and differential white blood cell (WBC) counts in healthy volunteers.

## Materials and Methods

**Study materials**

Carvacrol of pharmaceutical grade (90%) was purchased from Ji’An HaiRui Natural Plant Co. (China). Pellets were prepared by coating nonpareil beads (850–1180μm) with carvacrol using fluidized bed coater (Wurster insert, Werner Glatt, Germany). Then, 80% (w/v) of carvacrol was prepared by dispersing 5% hydroxypropyl methylcellulose (HPMC) and 2% Talc in absolute ethanol. This suspension was passed through a 140 mesh sieve. The suspension was sprayed into nonpareils beads using fluidized bed coater. The suspension was stirred throughout the layering process. Carvacrol layering process was carried out to produce pellets with about 7.5 and 11.75% (w/w) carvacrol load. After coating, the pellets were re-coated with HPMC 5% solution, fluidized for about 5 min and then, kept in an oven at 40^◦^C for 2 hr. The amount of carvacrol in pellets was measured by a gas chromatography (GC) method (Liolios et al., 2009). The GC analysis was performed using a Varian CP-3800 equipped with flame ionization detector (FID), fused-silica column (CP-Sil 8CB, 50 m × 0.25 mm, film thickness 0.12 μm). 

**Estimation of the stability of the products (pellets)**

Although all medicines were consumed through 3 months, an accelerated stability study (40°C±2°C/75% RH±5% RH) was carried out during a period of 6 months. The results showed no significant changes during this period (Sanjay et al., 2012 ▶). According to the ICH Q1 report from stability condition for world health organization (WHO) member states by region, Iran is categorized as IV A (hot and humid climate), so the shelf life for the products was found to be around 12 months. 

**Subjects**

The study was conducted at Department of Physiology of Mashhad University of Medical Sciences. Study protocol was approved by the ethics committee of Mashhad University of Medical Sciences and the trial was registered in Iranian Registry of Clinical Trial (IRCT No. IRCT2016080429191N1). In the present study, a total of 30 healthy male and female subjects (De Moraes et al., 2008 ▶; Mesia et al., 2011 ▶; Akrom and Darmawan, 2017 ▶) were recruited and signed consent forms were collected before enrollment. The subjects were randomly allocated to two groups by block randomization as block size was 4 and blocks were selected by random numbers table. Therefore, each group was comprised of 15 subjects. Volunteers were included if they complied with a number of inclusion and exclusion criteria. 

Inclusion criteria were being 20-40 years old, and having physical and mental health with no history of serious illnesses. In addition, lung examination was carried out in all healthy subjects and volunteers who entered the study. In this study, the FEV_1_ ranged from 80 to 115% in most of the volunteers, but in a number of subjects, it ranged between 78 and 80%. Study exclusion criteria were having cardiovascular diseases, brain and vascular diseases, malignancies, chronic inflammatory diseases, autoimmune disorders, psychiatric disorders, metabolic diseases, endocrine disorders, pregnancy, addiction to smoking or using any substances or taking any drugs. 

**Experimental protocol**

This study was a non-blind, randomized, dose-escalating study performed in the absence of control and placebo-treated groups. The study consisted of two groups, one group was treated with 1 mg/kg/day and the other group was treated with 2 mg/kg/day carvacrol capsules for 1 month. Venous blood samples were collected and PFT were performed in baseline and after the administration period. In addition, subjects were asked to report clinical signs and changes during the study. Carvacrol doses were determined based on 0.1 of the highest safe doses reported by animal studies (Jalali et al., 2013 ▶; Gholami Mahtaj et al., 2015 ▶; Reagan-Shaw et al., 2008 ▶; Shin et al., 2010 ▶).

**Biomedical measurements**

Pre and post-treatment levels of total and differential WBC counts as well as PFT values were recorded. Blood was analyzed for total WBC count and differential WBC including the percentages of lymphocytes (Lymph %), neutrophils (Neut %), eosinophils (Eos %), monocytes (Mono %) and basophils (Baso %). For this purpose, 1 mL blood was collected from each subject. WBC count analysis was done by an auto analyzer. Differential WBC counts were analyzed using stained peripheral blood smear (Wright-Giemsa) under a light microscope.

Spirometry was performed using a Spiro Analyzer (ST-90, Fukuda, Sangyo CO Ltd., Japan). PFT was first demonstrated by the operator, and PFTs were measured in a sitting position while wearing nose clips. PFT was done three times and the best values were chosen. The highest level of forced vital capacity (FVC), forced expiratory volume in the first second (FEV_1_), peak expiratory flow (PEF), maximum midexpiratory flow (MMEF), and maximal expiratory flow 25-75% (MEF25-75%), were recorded. This trial was done from January 15, 2016 to March 15, 2017.

**Statistical analysis**

Data was expressed as mean±standard deviation. Changes in variables after 1 month treatment in proportion to the baseline values, were calculated using the following equation;

Calculation of percent change of values post-treatment relative to pre-treatment;

$\frac{(\mathrm{post}-\mathrm{treatment value}-\mathrm{pre}-\mathrm{treatment value})\times 100}{\mathrm{pre}-\mathrm{treatment value}}$

Kolmogorov Simonov test was performed to determine distribution normality of data. Comparison between pre- and post-treatment values was made by the paired t-test. Two-independent sample test was used to compare variable changes between two groups during the treatment period. A p-value <0.05 was considered significant.

## Results

**Demographic characteristics of the study population**

Thirty healthy subjects including 9 males (30%) and 21 females (70%) with a mean (±SD) age 29.87 (±4.8) years, body weight 68.78 (±14.43) Kg, height 167.20 (±7.46) cm, and BMI of 24.49 (±4.12) Kg/m^2^ participated in the present trial. 

**Figure 1:**
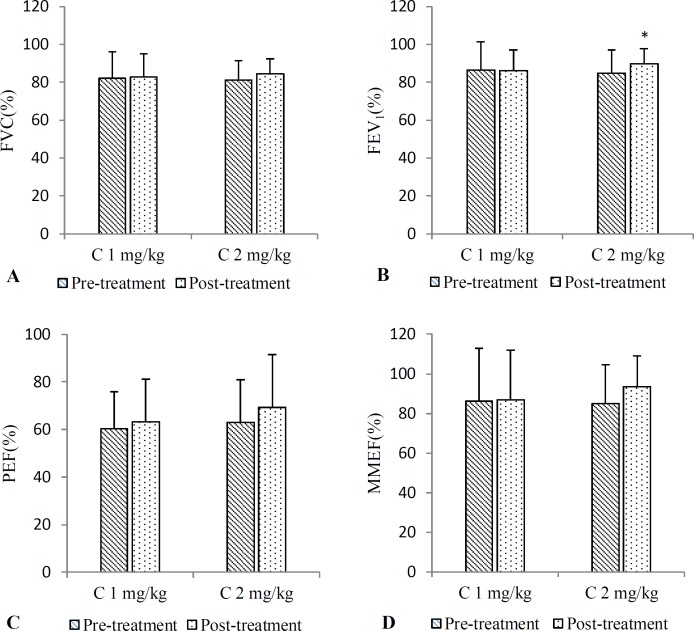
Pre and post-treatment PFT values including FVC (A), FEV_1_ (B), PEF (C), MMEF (D), in groups treated with low (C 1 mg/kg) and high (C 2 mg/kg) doses of carvacrol for one month (n=15 in each group). Values are expressed as mean±SD. *p<0.05 shows significant differences as compared to pre-treatment value. Statistical analyses were performed using paired sample t-test

FVC: forced vital capacity; FEV_1_: forced expiratory volume in first second; PEF: peak expiratory flow; and MMEF: maximum midexpiratory flow.

**Table 1:** WBC parameters recorded before (pre-treatment) and after (post-treatment) carvacrol administration for 1 month

	**C 1 mg/kg**		**P-value**	**C 2 mg/kg**		**P-value**
	**Pre-treatment**	**Post-treatment**		**Pre-treatment**	**Post-treatment**	
**Total WBC**	6.11±1.28	6.12±1.40	0.945	6.40±1.27	6.44±1.43	0.874
**LYMPH %**	41.21±8.89	39.26±8.57	0.358	42.50±7.56	43.40±7.58	0.519
**NEUT %**	46.97±9.06	48.04±9.18	0.542	41.13±7.30	41.57±7.67	0.802
**EOS %**	2.47±1.40	2.70±1.38	0.560	3.15±1.93	2.75± 1.21	0.472
**MONO %**	4.78±2.11	3.70±1.45	0.092	4.60±1.79	3.85±1.23	0.118
**BASO %**	1.19±1.14	1.17±1.08	0.922	0.90±1.07	1.00±1.09	0.908

Values are presented as mean±SD; n=15 in each group. Statistical analyses were performed using paired sample t-test.

LYMPH: Lymphocytes; NEUT: Neutrophils; EOS: Eosinophils; MONO: Monocytes; and BASO: Basophils.

**Effect of carvacrol on total and differential WBC counts**

Total and differential WBC counts of the two groups of carvacrol-treated subjects are provided in Table 1. Based on our data, no significant difference was observed between pre- and post-treatment values in both groups. 

**Effect of carvacrol on PFT parameters**

**Figure 2:**
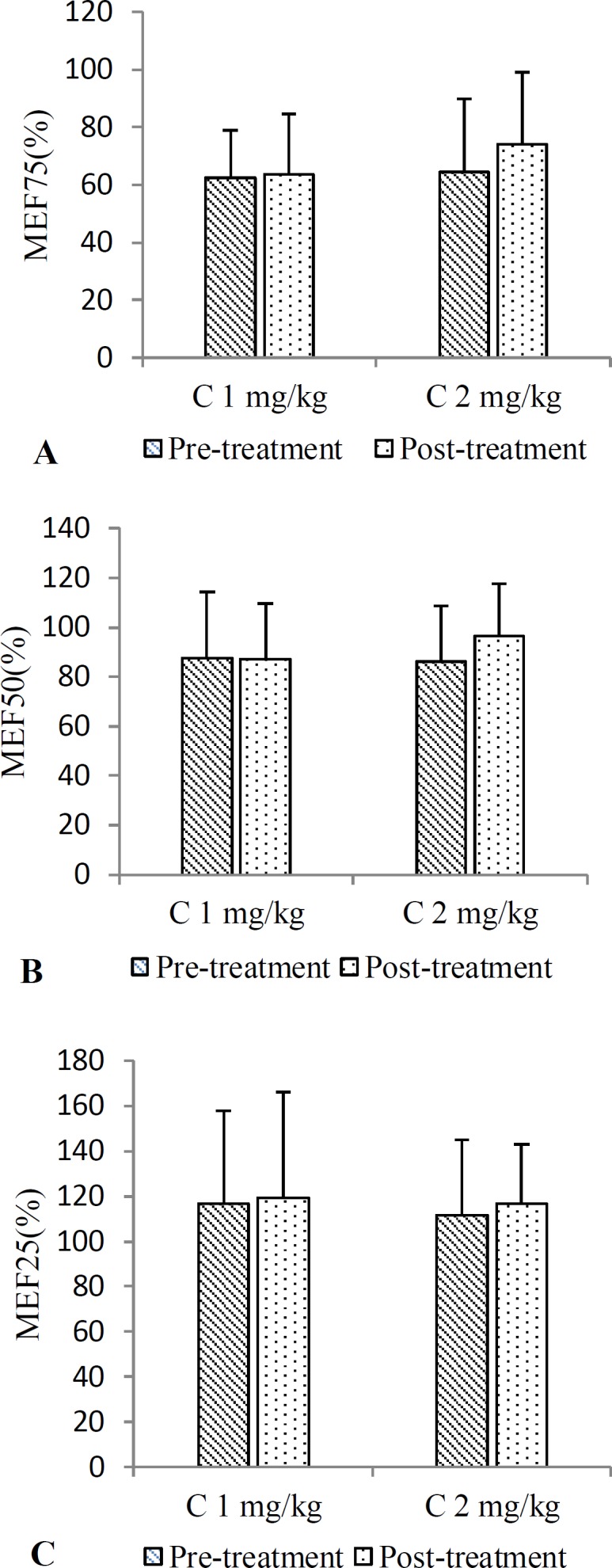
Pre and post-treatment PFT values including MEF75 (A), MEV50 (B), and MEF25 (C) in groups treated with low (C 1 mg/kg) and high (C 2 mg/kg) doses of carvacrol for one month (n=15 for each group). Values are presented as mean±SD. Statistical analyses were performed using paired sample t-test

There were no significant differences with respect to all PFT parameters (FVC, FEV_1_, PEF, MMEF and MEF25-75%) between pre- and post-treatment values in both groups. Only, post-treatment FEV_1_ value in group treated with higher dose of carvacrol was significantly increased compared to its pre-treatment value (p<0.05), (Figures 1 and 2).

MEF25-75%: maximal expiratory flow 25-75%.

**Figure 3:**
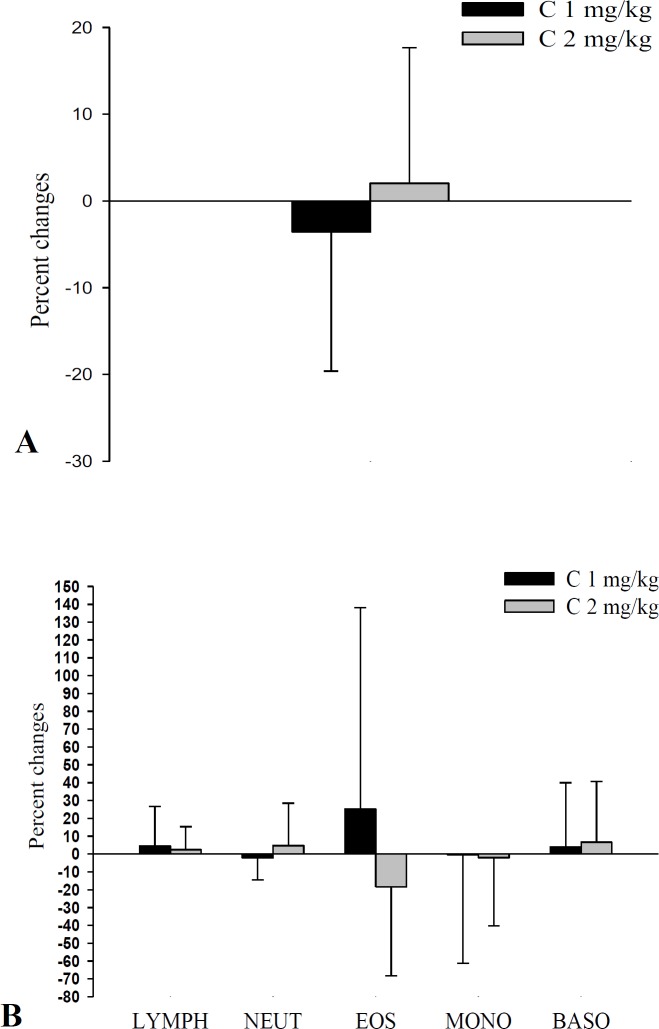
Percentage of changes (Δ) in total (A) and differential WBC (B) following 1-month treatment with two doses of carvacrol (n=15 in each group). Values are expressed as mean±SD. Statistical analyses were performed using Two Independent Sample t-test

**Figure 4:**
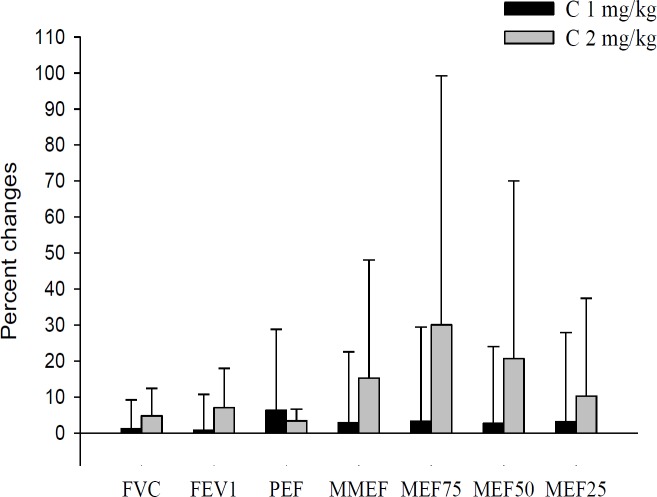
Percentage of changes (Δ) in PFT values following 1-month treatment with two doses of carvacrol (n=15 in each group). Values are expressed as mean±SD. Statistical analyses were performed using Two Independent Sample t-test

**Comparison of changes in WBC counts and PFT parameters between the two groups**

The changes in WBC and PFT parameters after 1-month treatment were comparable in both treatment groups and there were no significant differences in changes in PFT values, and total and differential WBC following the treatment period, between the groups (Figures 3 and 4).

## Discussion

This is the first report regarding the effect of carvacrol on total and differential WBC counts as well as PFT parameters in healthy humans. The results of the present study indicated that 1-month treatment with 1 and 2 mg/kg/day carvacrol did not affect PFT, total and differential WBC counts in healthy volunteers.

In fact, administration of carvacrol resulted in a small improvement of all PFT values in treatment groups which was statistically significant only for FEV1 values in the group treated with higher dose of carvacrol. In addition, both doses of carvacrol could not significantly change total and differential WBC counts. 

Carvacrol is found in a number of aromatic plants (Boskabady and Jalali, 2013 ▶) used in food at low levels (Suntres et al., 2015 ▶). Various biological and pharmacological effects including vasorelaxant, anti-inflammatory, and antioxidant effects were reported for carvacrol (Jayakumar et al., 2012 ▶; Landa et al., 2009 ▶; Boskabady and Jandaghi, 2003 ▶).

Carvacrol pharmacokinetics have been studies in animal models and data on carvacrol pharmacokinetics in humans are lacking. Only a single study was conducted on the pharmacokinetics of thymol (a carvacrol isomer) in humans (Kohlert et al., 2002 ▶). Based on these studies, after administration of carvacrol and thymol, these compounds are metabolized and found in the form of sulfates and glucuronides in urine and plasma. These compounds have a rapid metabolism and a short half-life (Michiels et al., 2008 ▶). 

In addition, studies that evaluated toxicological effects of carvacrol in animals, reported median lethal dose (LD_50_) of carvacrol. In this context, carvacrol administered intravenously to dogs had an LD_50 _of 0.31 g/kg and carvacrol administrated intra-dermally to rabbits had an LD_50 _of 2700 mg/kg (Livingston, 1970 ▶; McOmie et al., 1949 ▶). In addition, LD_50_ values in rats and mice (following intravenous administeration) were 810 mg/kg and 80.00 mg/kg of body weight, respectively (Hagan et al., 1967 ▶; Andersen, 2005 ▶). Inhibitory effect of 0.30 mM carvacrol on HepG2 cells was also reported (De Vincenzi et al., 2004 ▶; Stammati et al., 1999 ▶). However, there was no report for probable toxicological effects of oral carvacrol in humans. 

Also, some animal studies examined the effects of carvacrol on animals of both sexes. The effect of carvacrol on systemic and lung inflammation as well as oxidative stress in a guinea pig model of COPD in both sexes, was examined and the results showed a preventive effect for carvacrol on all measured parameters (Boskabady et al., 2015 ▶; Mahtaj et al., 2015 ▶). 

The present study, for the first time, examined the effects of carvacrol on PFT and WBC count in healthy volunteers of both sexes. The results of the present study showed that gender did not significantly affect various measured parameters. In addition, the results of this study showed no adverse effect following 1-month treatment with two doses of carvacrol in terms of PFT values, and total and differential WBC counts in healthy subject. Interestingly, carvacrol had a beneficial effect on PFT values. Therefore, according to the results of the present study, carvacrol might be considered safe when consumed for one month. 

However, the results of this study is not sufficient to confirm safety of carvacrol and further studies are needed in this regard. A number of questions remains to be answered in further studies. For example, the effect of higher doses of carvacrol or longer treatment-period on the above-mentioned parameters should be evaluated. Also, possible beneficial effects of carvacrol on lung function tests should be tested in respiratory diseases.

 Further studies should evaluate the effect of this agent on various diseases including asthma and COPD.

The results of this study showed that administration of carvacrol for one month to healthy humans, does not cause any clinically significant changes in measured parameters. Therefore, carvacrol can be used in further researches in healthy humans and patients with various diseases especially obstructive respiratory disorders.
